# Heat shock protein-70 is elevated in childhood primary immune thrombocytopenia

**DOI:** 10.1186/s40001-022-00868-9

**Published:** 2022-11-11

**Authors:** Jiao Ge, Yan Liu

**Affiliations:** 1 Emergency Department, West China Second University Hospital, Sichuan University, No.20, Section 3 of Renmin South Road, Wuhou District, Chengdu, 610041 China; 2 Key Laboratory of Birth Defects and Related Diseases of Women and Children, Ministry of Education, Sichuan University, Chengdu, China

**Keywords:** Childhood immune thrombocytopenia, Heat shock proteins, HSP-70, Platelets

## Abstract

**Background:**

Immune thrombocytopenia (ITP) is an acquired autoimmune disorder characterized by the destruction of the platelets resulting from autoimmune recognition and subsequent attack. Heat shock proteins (HSPs) are directly associated with progression and pathogenesis in some specific autoimmune diseases. The aim of this study was to investigate the serum expression of HSP-70 in ITP children and healthy controls.

**Materials and methods:**

A total of 86 children aged 1–6 years were enrolled in the study. The participants were divided into 20 newly diagnosed ITP (ndITP), 34 chronic ITP (cITP) patients and 32 healthy children. The white blood cells and platelet counts were determined and compared between the groups. HSP-70 serum levels were analyzed by sandwich ELISA. Data analysis was done using SPSS and the data variables assessment was done through histogram, probability plots and Shapiro–Wilk tests to determine normal distribution.

**Results:**

The white blood cell counts were 8.9 (4.2–10.4) for new diagnosis ITP, 7.1(3.9–11.9) for the chronic ITP group and 7.0 (4.3–9.5) for the healthy controls. The platelet counts were significantly increased in the chronic ITP group, 83.5(31.7–297) compared to the ndITP group 27.4 (3.7–63.7), but significantly lower compared to the healthy controls 271(172–462) (*P* = 0.0009). There were significantly increased HSP-70 serum levels in cITP patients compared to the ndITP and the healthy group. In addition, there was a positive correlation between the serum HSP-70 level and the thrombocyte counts among the ITP children.

**Conclusions:**

HSP-70 has a role in the progression of childhood ITP. Increased HSP-70 level is associated with the severity of childhood primary ITP.

## Background

Immune thrombocytopenia (ITP) is described as an acquired autoimmune disorder characterized by the destruction of the platelets as a result of autoimmune recognition and subsequent attack. By definition, ITP is a platelet count below 100 × 10^9^/L in the absence of any non-immune or immune diseases that may lead to thrombocytopenia [1]. The classical characteristic of ITP is the opsonization of platelets by immunoglobulin G auto-antibodies and the subsequent demolition of these complexes by the Fc receptor-mediated phagocytosis through macrophages occurring in the spleen [2]. In addition, other modes of thrombocytopenia mediated by the immune system exist, such as the T cell-mediated cytotoxicity, which could result in the down-regulation of the megakaryocytes in the bone marrow leading to deficiency in the production of platelets [3].

ITP peaks between 1 and 2 years. Even though ITP has no age limit from infancy to adulthood, girls and boys receive equal treatment in childhood. In children, ITP mostly presents itself in a self-limiting manner for a maximum of 12 months in the presence or absence of treatment, and the platelet counts may eventually return to normalcy [4]. Nevertheless, in about 20% of the newly diagnosed childhood cases, the ITP develops to the chronic form defined based on the standardization criteria [5]. Chronic ITP presents with purpura, petechial and the bleeding of the mucosa that is often due to the infection of the upper respiratory tract. The important biomarkers to define children who may develop chronic ITP disease forms are partially unavailable.

Heat shock proteins (HSPs) are described as highly conserved protein groups whose classification depends on their molecular weight and are generated by prokaryotic and eukaryotic cells in both normal and stressful environments [6]. The HSPs occur in reduced concentrations in the normal and unstressed cells, where they have essential physiological functions, including acting as chaperones to help in the proper polypeptides folding and assembly, intracellular transportation of other molecules between various compartments within the cells and the processing of MHC and peptide complexes. According to the current evidence, stress protein may be a crucial factor in the etiology and pathogenesis of different autoimmune infections, such as immune thrombocytopenia [7].

Upregulated HSP-70 levels and HSP-70 autoantibodies in sera of juvenile idiopathic arthritis (JIA) or rheumatoid arthritis (RA) patients have been linked with the progression and activity of the diseases [8]. According to the various in vitro investigations, HSP-70 has pro-inflammatory properties during their interactions with innate immune cells. Contradictory to the confirmed pro-inflammatory characteristics of HSP-70, literature also indicates that HSP-70 might have great anti-inflammatory functions, and the correlations between disease activity and the expression of HSP-70 might be an accompanying situation [9]. However, the association between HSP-70 and childhood primary Immune thrombocytopenia requires further investigation. Here, the association between HSP-70 and childhood ITP is are explored.

## Materials and methods

### Patients and ethical statements

This study enrolled 54 ITP children (aged 1–6 years, mean) diagnosed in the Emergency Department, West China Second University Hospital, Sichuan University from August 2020 to November 2021. Thirty-two healthy children between 1 and 6 years were also enrolled in the study as controls. The diagnosis was done following the International Working Group (IWG) consensus criteria based on the exclusion of other infections leading to thrombocytopenia, including autoimmune diseases. As stipulated by the IWG, patients diagnosed within the previous 3 months were regarded as newly diagnosed immune thrombocytopenia (ndITP), and children with ITP diagnosed over 12 months were regarded as chronic ITP (cITP). Twenty out of the 54 ITP children were reported as the new diagnosis (ndITP), while 34 children were reported to be in the chronic ITP (cITP) phase. The patients' management was done following the IWG consensus. Age, sex and complete blood count data were obtained during the study and control group enrolment. The ITP and healthy controls' characteristics are presented in Table 1. The study was authorized by the Ethical Review Board of West China Second University Hospital, Sichuan University under the ethical number Kx082. Written consent was obtained from the guardians of all the children included in the study.

**Table 1:** Comparison of the clinical characteristics of the ITP children and the healthy controls

	ITP		Controls	*P*
	and ITP	cITP	Healthy controls	
Total (n)	20	34	62	
Age, median: min–max	2 (1–5)	5(2–6)	4 (1–6)	0.423
Sex: female/male	14/6	22/12	22/10	0.721
WBC (× 10^3^/uL) Min–Max (median)	8.9 (4.2–10.4)	7.1 (3.9–11.9)	7.0 (4.3–9.5)	0.063
Plt (9 × 10^8^/L); Min–Max (median)	27.4 (3.7–63.7)	83.5 (31.7–297)	271 (172–462)	0.0009*
Hb (g/dl); Min–Max (Median)	12.7 (11.4–15.78)	12.4 (11.6–16.0)	12.6(11.9–16.1)	0.711

*cITP *chronic ITP, *ndITP *newly diagnosed ITP, *Hb *hemoglobin, *plt *platelets, *WBC *white blood cells

*P < 0.005

### HSP-70 measurement through ELISA

The blood samples were obtained from both the ITP and the control groups. To obtain serum, the blood samples were centrifuged for 10 min at 3000 g and 4 °C. The samples were then processed and quickly frozen, and kept at − 80 °C for use in the subsequent experiments. During the sampling time, all the patients had not received any treatment from immunosuppressive drugs for approximately 3 months. The Serum Hsp-70 were assessed using commercial ELISA kits (Thermo-Fisher Scientific, Waltham, MA, USA), following the manufacturer's instructions. Briefly, the human monoclonal antibody was first pre-coated to the 96-well culture plate. The plate was later incubated for 2 h at room temperature to enable the HSP-70 to bind to the immobilized antibody. Next, the plate was washed to eliminate the unbound antibody. Later, Hsp-70-specific enzyme-conjugated antibody was in each well. The plates were then washed to remove the unattached enzyme and enzyme reagent. A substrate was added to each well to allow for the development of a color, proportional to the bound HSP-70. The colored reaction intensity was determined at 450 nm with an automated ELISA reader. Expression of the results was done in the form of ng/ml.

### Statistical analysis

Data analysis was done using IBM SPSS 26.0 (SPSS Inc, Chicago, IL) software. Assessment of the data variables was done through histogram, probability plots and Shapiro–Wilk tests to determine normal distribution. Non-parametric analyses were performed in case the variables were not normally distributed. Median values were reported with minimum–maximum values to keep with the non-parametric tests. Multi-group analyses were done using the Kruskal–Wallis test, while the non-parametric variables between the two groups were compared using the Mann–Whitney *U* test. While assessing the associations between thrombocyte counts and the levels of HSP, the correlation coefficients and their significance were analyzed through the Spearman test. The results were considered significant when *P* < 0.05.

## Results

In total, 54 children with ITP and 32 healthy controls were included in this study. The clinical features of the patients are presented in Table 1. Twenty children were newly diagnosed ITP cases, while 34 children had chronic ITP. In the newly diagnosed ITP (ndITP), 14 (70%) were female, while 6(30%) were males. In the chronic ITP (cITP), 22(64.7%) were female, while 12 (35.3%) were males. There was no significant difference in the sexes between the groups (*P* = 0.721). The ndITP patients also demonstrated higher age in comparison with the cITP patients. The white blood cell counts were 8.9 (4.2–10.4), 7.1 (3.9–11.9) and 7.0 (4.3–9.5) for new diagnosis ITP, chronic ITP and healthy controls, respectively. Furthermore, the platelet counts were significantly increased in the chronic ITP group (83.5 (31.7–297) compared to the ndITP group 27.4 (3.7–63.7), but significantly lower compared to the healthy controls 271 (172–462) (*P* = 0.0009).

As shown in Table 3, the initial bleeding score was remarkably elevated in the cITP compared to the ndITP group (*P* = 0.005). The immature platelets fraction (IPF) was also significantly elevated in the cITP compared to the ndITP (*P* < 0.001). However, we observed no significant difference regarding other laboratory parameters, including MPV (FL), PDW, P-LCR and PCT (%) (Table 2).

**Table 2:** Parameters of platelets between the new diagnosis and chronic ITP children

Variable	ndITP *n* = 20	cITP *n* = 34	*P* value
Initial bleeding score			
Mean ± SD	3.9 ± 1.1	5.7 ± 2.1	0.005
Range	2.5–9	4–10	
Platelets (× 10^9^/L)			
Median (IQR)	116 (117)	33.3 (52.4	0.013
Range	6–144	5–141	
MPV (FL)			
Median (IQR)	10.6 (2.1)	10.8 (2.0)	0.052
Range	7.4–14.1	9.3–14.0	
% P-LCR			
Mean ± SD	37.4 ± 14.1	40.7 ± 12.3	0.412
Range	27.6–57.1	33.5–61.3	
PDW (FL)			
Mean ± SD	13.6 ± 3.1	14.9 ± 2.1	0.21
Range	10–19.1	12.4–17.5	
% IPF			
Median (IQR)	10.3 (13.6)	20.1 (17.5)	< 0.001
Range	3.1–29.5	3,2–40.2	
% PCT			
Median (IQR)	0.2 (0.09)	(0.08)	0.399
Range	0.02–0.19	0.03(0.18	

Data expression is done as mean SD. Comparison was done using the student’s *t* test

*MPV *mean platelet volume, *P-LCR* platelet large cell ration, *PDW *platelet distribution width, *IPF *immature platelet fraction, *PCT *plateletcrit

**Fig. 1:**
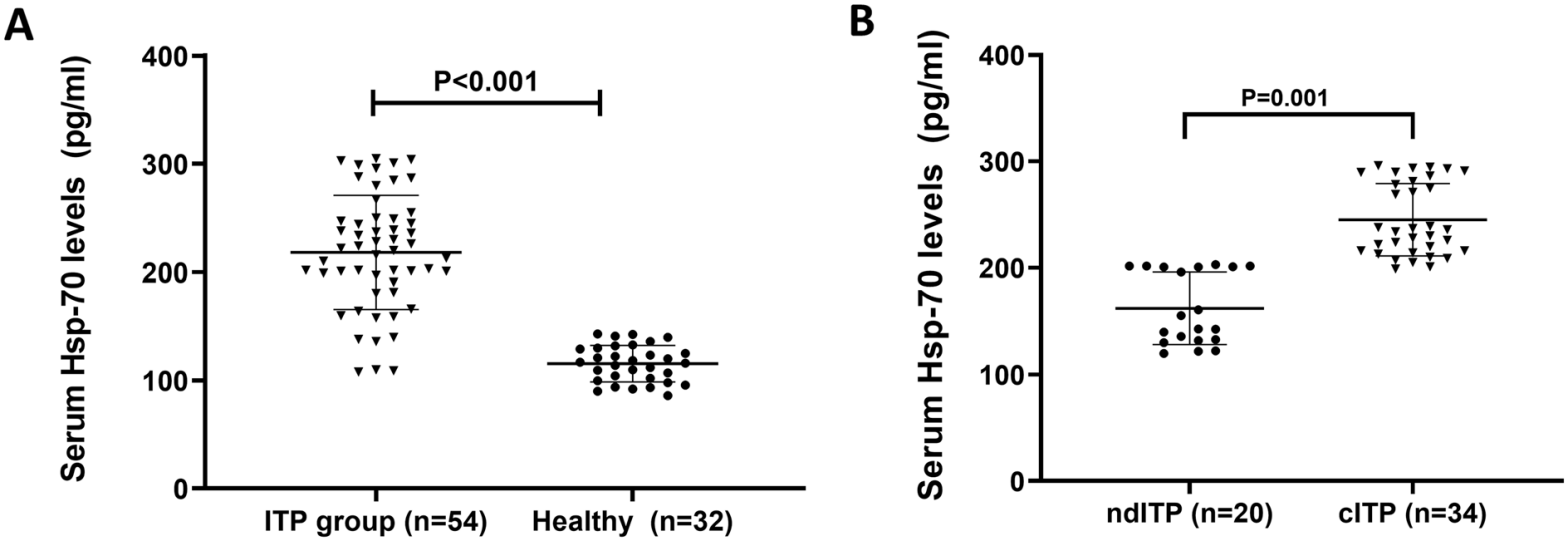
Assessment of Hsp-70 expression in new diagnosis and chronic ITP. **A** The levels of Hsp-70 in the serum of the ITP patients (n = 54) and the healthy group (control) (n = 32). **B** the Hsp-70 levels in the serum of patients with stages ndITP (n = 20) and cIPT (n = 34). The data expression is in the form mean ± SD

The level of expression of HSP-70 in patients with ITP and the healthy controls was determined through the sandwich ELISA. According to our results, the serum samples from the ndITP patients contained significantly increased HSP-70 levels (186.1 pg/ml (136.4–202.6) compared to the healthy controls (116.61 pg/ml (86.0–143.0). The Hsp-70 serum levels in cITP patients (244.5 pg/ml (203.4–310.3) were also significantly higher compared to the healthy controls *P* < 0.001, as shown in Table 3. Similarly, the serum HSP-70 levels were investigated between the various groups, and the results were analyzed. The observations confirmed that the mean HSP-70 levels in the ITP group were 218.2 ± 7.168 95% CI 108–305, while the mean HSP-70 level in the healthy group was 115.6 ± 2.982 95% CI 109.5–121.6 *P* = 0.001, as shown in Fig. 1A. On the other hand, analysis of the expression of the serum levels between the ndITP and cITP revealed that the ndITP had a mean serum level of 162.2 ± 7.566, 95% CI 146.4–178.0, while the mean serum level of the cITP group was 245.1 ± 5.84, 95% CI 233.2–257.0, *P* = 0.001 as shown in Fig. 1B.

**Table 3:** Median Hsp-70 levels in the serum of ITP patients versus the healthy control group

	ndITP (*n* = 20)		cITP (*n* = 34)		Control (*n* = 32)		*P* value
	Median	Range	Median	Range	Median	Range	
Hsp-70 level in serum (pg/mL)	186.1	136.4–202.6	244.5	203.4–310.3	116.61	86.0–143.0	< 0.001

**Fig. 2:**
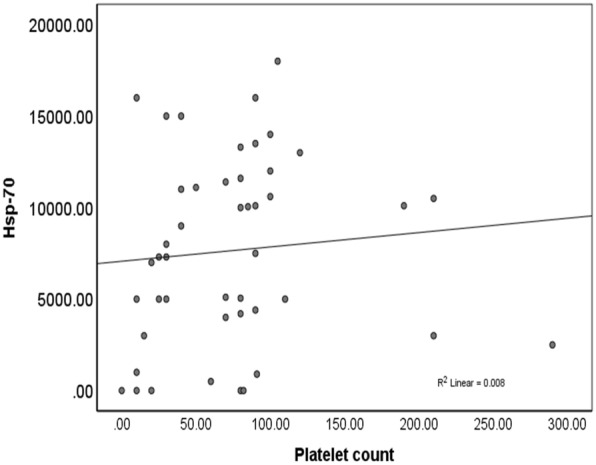
Spearman correlation assessment between the levels of Hsp-70 and platelets counts in the ITP children

The ndITP and cITP patients having thrombocyte counts below 30 × 10^9^/L demonstrated significantly inhibited levels of HSP-70 compared to patients with the number of thrombocytes exceeding 30 × 10^9^/L. In addition, a positive correlation was noted between the levels of HSP-70 and the number of thrombocytes, confirming an increase in the level of HSP-70 with the rise in the counts of thrombocytes, as shown in Fig. 2.

## Discussion

The present study assessed the HSP-70 serum levels in newly diagnosed and chronic ITP and compared their expressions to the healthy controls. Our findings showed that the HSP-70 levels in newly diagnosed and chronic ITP children were significantly increased compared to the healthy control group. In addition, the chronic ITP group demonstrated significantly elevated HSP-70 levels compared to the newly diagnosed ITP group. In addition, a positive correlation was observed between the levels of HSP-70 and the thrombocyte counts.

HSP-70 has been reported to play varying roles in the progression of various inflammatory diseases. The molecule has been confirmed to regulate intracellular homeostasis and is released into the extracellular environment as a response to acute myocardial infarction (AMI) [10]. Increased circulating HSP-70 has been discussed as a myocardial damage marker post-AMI, which is linked with the inflammatory responses [11], and might also enhance the heart failure progression. Furthermore, HSP70 was significantly increased post-AMI and was directly linked to severe cardiac events [10]. However, some studies indicated that high HSP-70 levels might have athero-protective impacts [12], and exosomes from healthy humans might offer cardio-protection in cardiac ischemia and reperfusion through HSP70-mediated effects [13].

In a study involving asthmatic patients, remarkably elevated HSP-70 autoantibodies were reported to be associated with asthma. A significant positive correlation was further reported between HSP-70 and the severity of asthma symptoms [14]. Contrastingly, some studies also reported that HSP-70 plays an auto-protective role in lung injury and asthma. Furthermore, HSPs autoantibodies have been confirmed to have notable roles in the pathogenesis and prognosis of various other diseases. For instance, Shinghai and colleagues confirmed antibodies against HSPS in autoimmune liver disease patients and hypothesized that anti-HSP70 presence indicates the disease activity of primary biliary cirrhosis [14].

No literature has, however, reported the role of HSP-70 in the development of childhood immune thrombocytopenia. Certain parameters provide information about platelets, overall referred to as platelet indices, and they include the platelet distribution width (PDW), mean platelet volume (MPV) and the platelet large cell ratio (PLCR) [15]. In platelets, various HSP-70-linked genes have been specified for the trafficking and sequestration of proteins. Classical endoplasmic reticulum chaperone, BiP/Grp78, is one member of the HSP70 family that has been categorized in the cytosol of the platelet, in circulation and on the surface of the platelet, whereby it has antithrombotic function through pro-coagulant tissue factor sequestration on the surface of the platelet [16].

More recent findings have indicated that the platelet HSP90/Hsp70 system has been hypothesized to modulate intracellular trafficking and multidrug resistance MDR4/ABCC4 localization [17]. The HSP members additionally play essential roles in the assembly of actinomyosin filament, an important step in the change of platelet shape and spreading [18]. For instance, HSP27 has been implicated as a downstream p38 MAPK target in the assembly of the cytoskeleton, and phosphorylation of HSP27 is important for the secretion of platelet granules [19]. Taken together, these reports indicate the HSP70 intracellular and extracellular functions and associated chaperones in trafficking, assembly of protein and platelets regulation. In this investigation, Hsp-70 concentration was positively correlated with the platelets counts.

ITP has imbalances in pro- and anti-inflammatory mechanisms [20]. According to various studies, pro-inflammatory CD4 + T-helper 17 cells that generate interleukin 17 have a pathogenic function in an active autoimmune infection [21]. Contrastingly, regulatory T cells constituting 5–10% of the peripheral CD4 + T cells have an important function in self-tolerance, inhibit antibody- and cell-mediated immunity responses and protect the host from autoimmunity [22]. Among the most unique aspects of the physiology of T-regs is their opposing feature against pro-inflammatory Th17 cells and suppressing their autoimmune capability in a particular way [20]. According to Nishimoto et al., about 36% of the Tregs-deficient mice became thrombocytopenic for about 5 weeks. IgG antiplatelet antibodies were reported in these thrombocytopenic mice, and the transfer of purified T-regs into the experimental mice alleviated the thrombocytopenia [23].

At present, HSPs have been regarded as toxic autoantigens and have an association between autoimmunity and infection. Nevertheless, growing evidence has indicated that HSPs such as HSP-70 might demonstrate anti-inflammatory characteristics in some circumstances. HSPs that have anti-inflammatory properties, such as HSP-70, specifically affect the presence or the functional activities of these immune-regulatory cells, especially since these stress genes are reported ligands for toll-like receptors (TLRs) [24]. The TLRs are normally expressed on the regulatory T cells, and the TLRs ligation on CD4 + CD25 + T cells initiates a tenfold rise in their inhibitory activity [25].

Our findings show that HSP-70 is elevated in childhood ITP. This observation confirms that Hsp-70 can play an immunosuppressive role, which has also been reported by previous literature. As an immunosuppressant, HSP-70 has been shown to pass through a lipid bilayer, an ability-linked phosphatidylserine presence. Additional sphingolipids, including globotriaosylceramide also induce the insertion of HSP-70 into a membrane, confirming that HSP-70 might be present in the tumor membranes [26].

Myeloid-derived suppressor cells (MDSCs) are a heterogeneous cell population that expands during inflammation, cancer and infection, with a significant ability to suppress T cell responses [27]. According to Chalmin and colleagues' findings in humans and mice, membrane-related HSP-70 present in tumor-derived exosomes (TDEs) inhibited tumor immune regulations by enhancing the suppressive roles of MDSCs. TDEs contained in the supernatant of tumor cells of three cancer cell lines could mediate T cell-dependent immunomodulatory functions of MDSCs. It was identified that the TDEs factor that induced activation of MDSCs was the inducible TDE cell surface-expressed HSP-70 (HSPA1A) [27]. These observations indicated that immunosuppressive effects of tumor cells include their ability to induce functional MDSCs by producing exosomes that express HSP-70. The limitation of this study is that it did not investigate the HSP-70 expression in ITP patients' tissues and lacked in vivo studies to investigate the association of HSP-70 with other important inflammatory factors, such as TNF-α and IL-4. Furthermore, a longitudinal study is needed to determine whether levels of HSP-70 in ndITP change once they develop either chronic ITP or go into remission. Another limitation is the possibility that thrombocytopenia itself leads to higher levels of HSP-70 in circulation.

## Conclusions

This is the first investigation to report the extracellular HSP-70 level in childhood primary ITP. HSP-70 has a direct role in the inflammation and establishment of childhood ITP. This study provides a new insight into the use of HSP-70 as a biomarker to determine the establishment and progression of ITP in children.

## Data Availability

All the data generated during the investigation have been provided. However, should the raw data be needed, the authors are willing to provide after a written application.
